# Supplementary material for: Probing the zooarchaeological record across time and space for ancient pathogen DNA
Source: Nat Commun. 2026 Apr 30;17:3469. doi: 10.1038/s41467-026-71543-4 (PMC13133277; doi:10.1038/s41467-026-71543-4)
Supplement: Supplementary file 1 — Supplementary Information [file 41467_2026_71543_MOESM1_ESM.pdf]

Supplemental Online Material

Probing the zooarchaeological record across time and space for ancient pathogen DNA

Anne Kathrine W. Runge, Ian Light-Maka, Ken Massy, Marcel Keller, Simon Trixl, Helja Kabral, Casey L. Kirkpatrick, Kirsten Bos, Jana Eger, Michal Ernée, René Kyselý, Michael Hochmuth, Dominik Poradowski, Aleksander Chrószcz, Norbert Benecke, David Daněček, Jana Klementová, Anatoli Nagler, Alexey A. Kalmykov, Anatoly R. Kantorovich, Vladimir E. Maslov, Andrey B. Belinskiy, Christiana L. Scheib, Meda Toderaş, Svend Hansen, Philipp W. Stockhammer, Kai Kaniuth, Regina Uhl, Sabine Reinhold, Rosalind E. Gillis, Elizabeth A. Nelson, Kamilla Pawłowska, Felix M. Key

Supplementary Figures

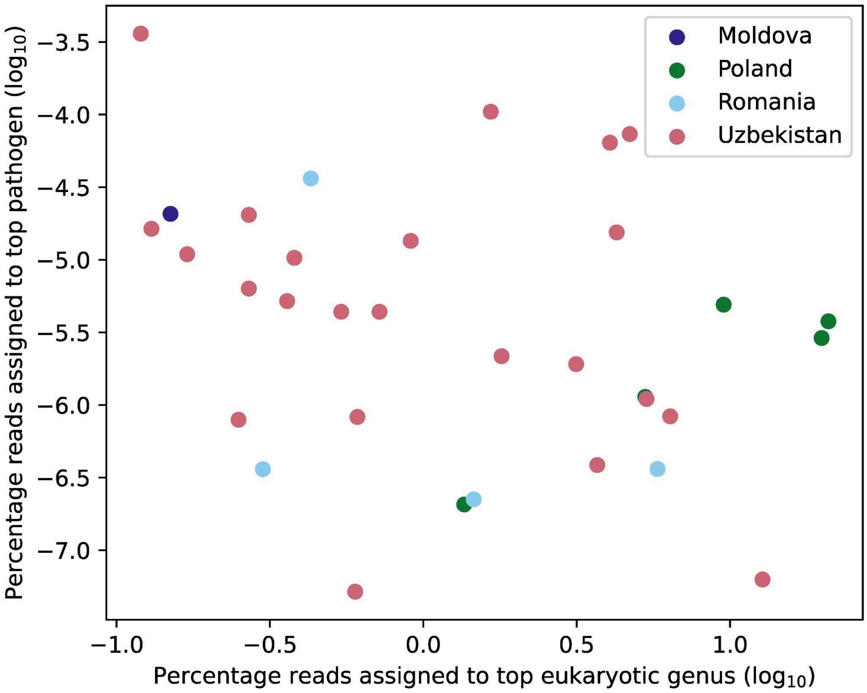

Figure S1. Percentages of reads assigned to top eukaryotic genus vs percentage of reads assigned to top pathogen species for samples with a pathogen identified. The pathogen with the highest number of reads was plotted for samples with multiple identified pathogens. No correlation is observed between pathogen and eukaryotic read percentages, suggesting that preservation of host DNA does not predict pathogen recovery (R^2=0.023)

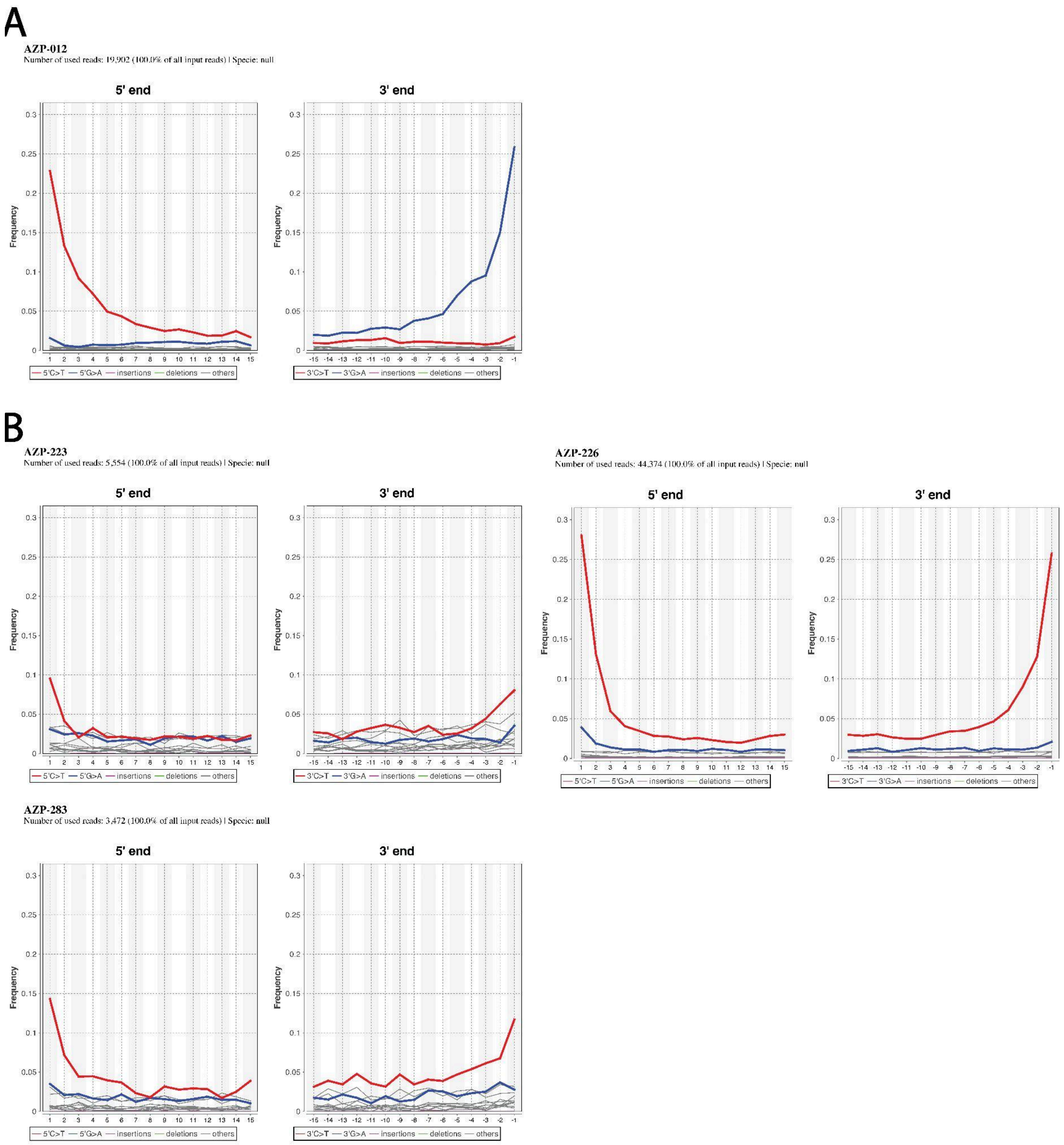

Figure S2. Damageprofiler plots display rates of base substitutions reflective ancient origin for A) *E. rhusiopathiae* and B) *S. lutetiensis*. AZP-012 was prepared using a double-stranded protocol and thus has C->T transitions on the 5' end and G->A transitions on the 3' end of reads. AZP-223, AZP-226, and AZP-283 display C->T transitions on both the 5' and 3' ends of the mapped reads, consistent with single-stranded library preparation.

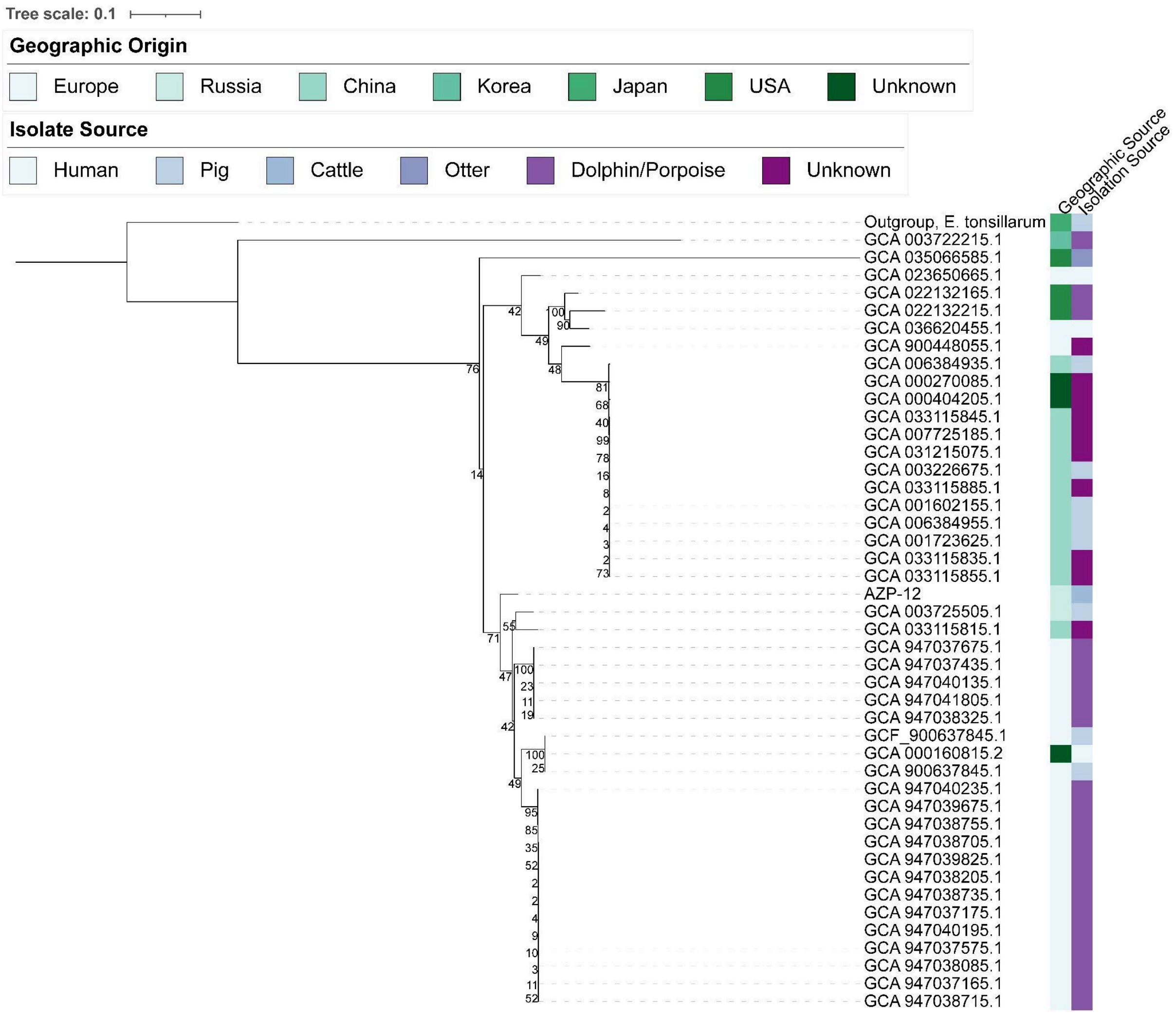

Figure S3. *E. rhusiopathiae* AZP-012 single sample phylogenetic placement. Previously published ancient genomes are excluded.

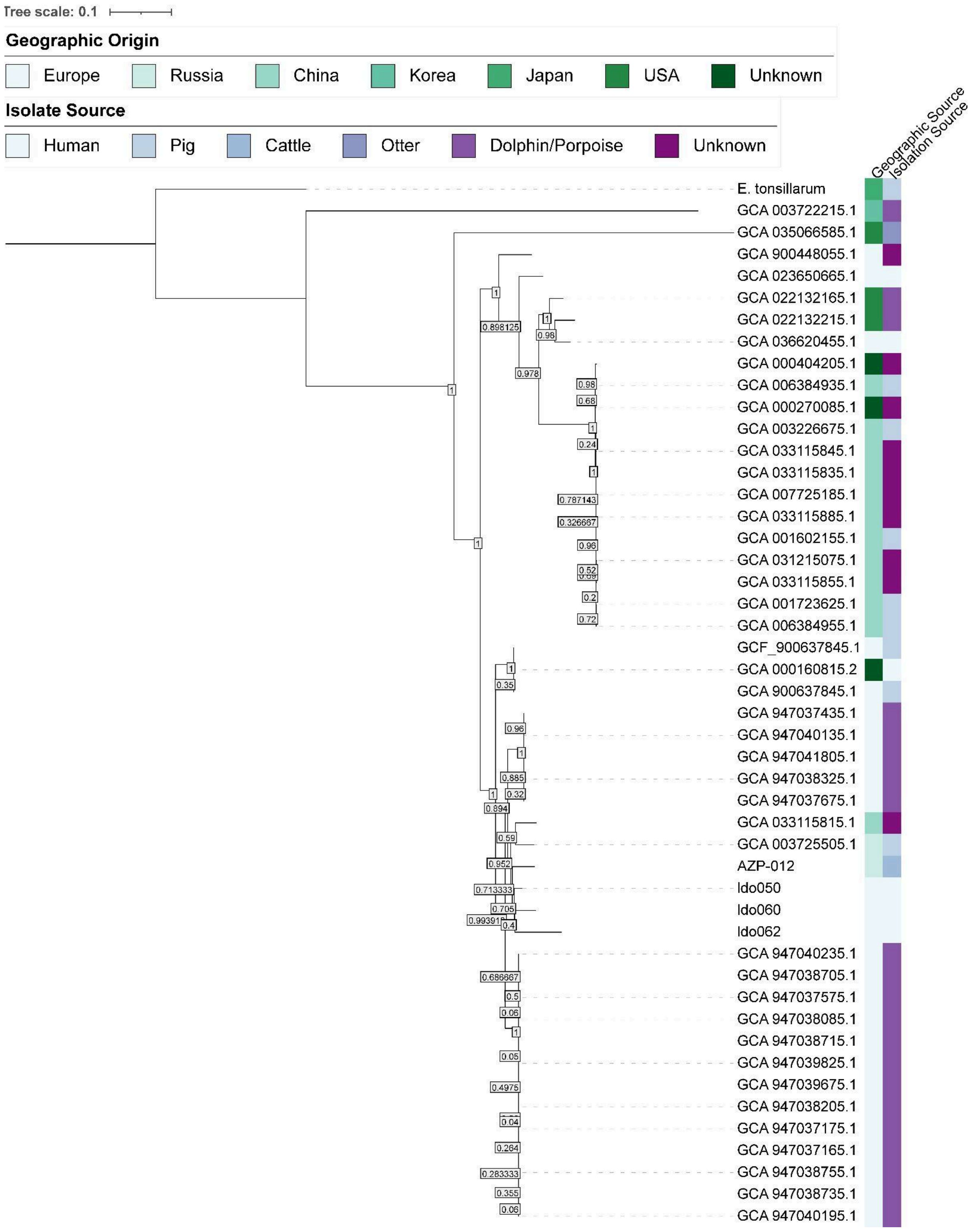

Figure S4. *E. rhusiopathiae* Transfer-Bootstrap-Expectation statistics. Bootstrap value of node separating ancient samples is 0.952, highlighted in bold.

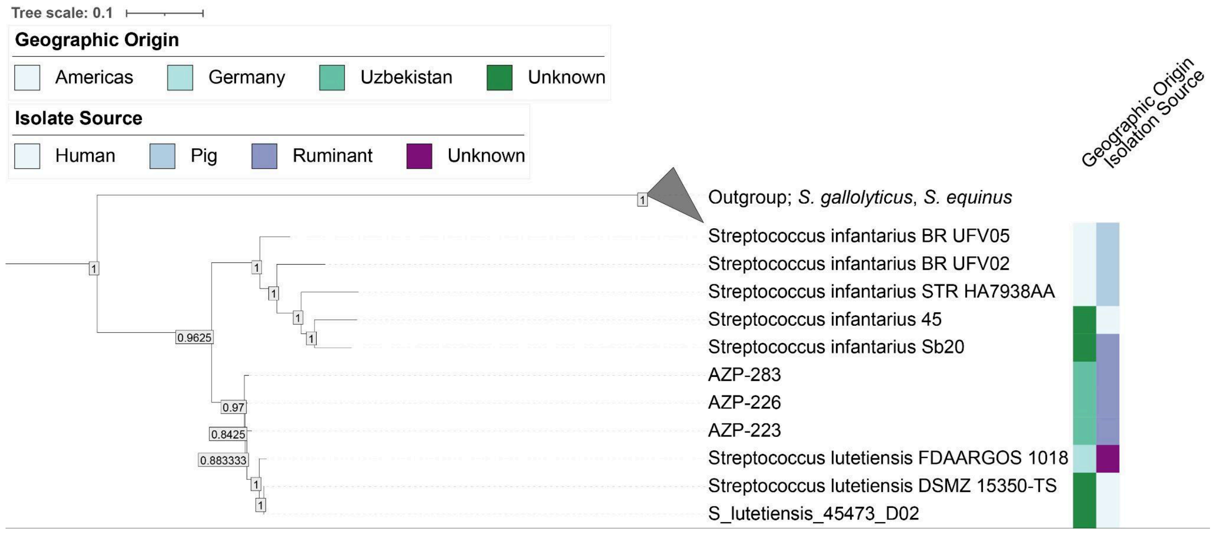

Figure S5. *S. lutetiensis* Transfer-Bootstrap-Expectation statistics. Bootstrap value of node separating ancient samples is 0.952, highlighted in bold.

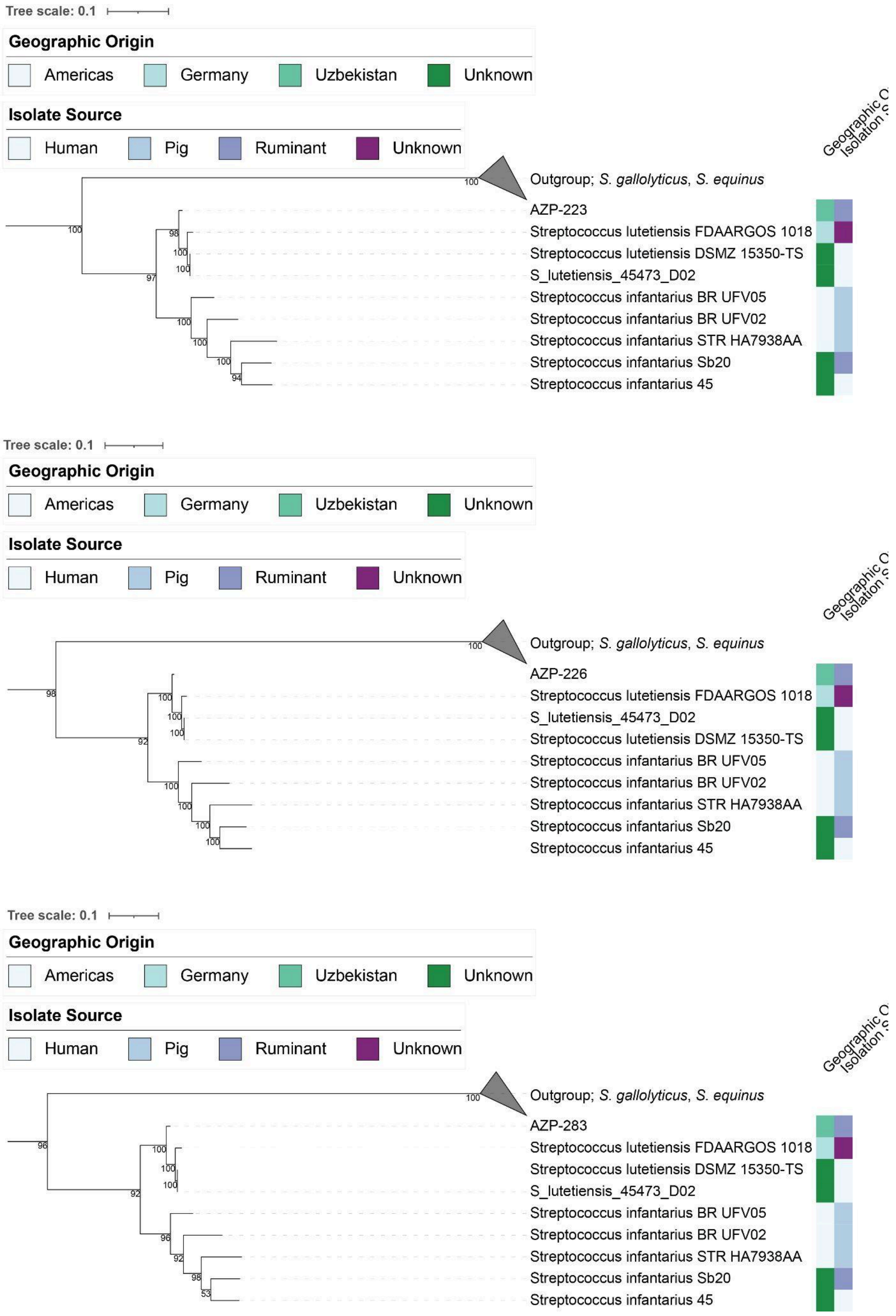

Figure S6. *S. lutetiensis* single sample phylogenetic placements.

Materials and methods

Site, fauna, and palaeopathology results

Ken Massy, Simon Trixl, Jana Eger, Michal Ernée, René Kyselý, Michael Hochmuth, Dominik Poradowski, Aleksander Chrószcz, Norbert Benecke, David Daněček, Jana Klementová, Anatoli Nagler, Alexey A. Kalmykov, Anatoly R. Kantorovich, Vladimir E. Maslov, Andrey B. Belinskiy, Meda Toderaş, Svend Hansen, Philipp W. Stockhammer, Kai Kaniuth, Regina Uhl, Sabine Reinhold, Rosalind E. Gillis, Kamilla Pawłowska

Czech Republic

Dolní Beřkovice, Czech Republic

The polycultural site located between Dolní Beřkovice and Křivenice (location Dýhárna Danzer; 50.40343°N, 14.43944°E) was excavated between 2001 and 2005 by the Institute of Archaeology of the Czech Academy of Sciences in Prague. It yielded a large number of animal bone remains. The sampled dog tooth (*caninus inferior sinister*) was excavated from grave 932 (ID: 5933) alongside a human skeleton laying on the left side, a position typical for females, belonging to the Corded Ware Culture (CWC) [1,2].

Hostěnice (site Brozany), Czech Republic

The site Brozany (Hostěnice) is located in lowlands between Central and North Bohemia (50.44265°N, 14.14778°E) and was excavated 1997 [3]. Osteological material [4] dated to the Funnelbeaker Culture represents standard settlement waste, but puppy skeletons and fox skeletons were also found. One dog (ID: 726) tooth (*premolar 3 inferior sinister)* was selected for DNA analysis in this study. The tooth, likely an offering, was found in settlement storage pit No. 181A.

Hostivice-Palouky, Prague-West district, Czech Republic

Contact persons: David Daněček, Jana Klementová

A rescue excavation at Hostivice-Palouky (50.57479°N, 13.68595°E) was carried out by J. Klementová and D. Daněček (Museum Roztoky) in 2007–2008 and 2010–2013 [5,6]. The excavated area covers 10 ha, more than 1,300 settlement pit features and a similar number of post holes were found (2626 features altogether). The site was occupied during the Linear and Stroked Pottery cultures, the Funnel Beaker and Řivnáč cultures, Hallstatt (Ha C-D1), Roman Iron Age, and Early Middle Ages. More than 330 graves were also uncovered, and burials/skeletons were also identified in sunken settlement features from the Funnel Beaker (Baalberge stage), Corded Ware, Bell Beaker and Knovíz cultures (B D – Ha A2), as well as from the La Tène period (Lt B1-C), Migration period, and Early to Late Middle Ages. A small group of graves, few other single graves, two settlement pits (1356/2008 and 1415/2008), as well as a group of few other sunken features (1723/2011) interpreted as

“ceremonial structure” and possible “foundation deposit” [7,8] were dated to the Bell Beaker Culture. Multiple human skeletons were sampled for ancient DNA and presented previously [9] and several human specimens positive for *Yersinia pestis* from grave 17/22 part of Hostivice-Palouky have been described [10]. Anthropological research was performed by M. Dobisíková (National Museum Prague). One pig and one cattle tooth, both from settlement pit 1356, were included in this study.

Libkovice, Czech Republic

Archaeological research carried out in 2018 by the Institute of Archaeological Monument Preservation of Northwest Bohemia in Most (V. Sušická) focused on the southeastern part of the cadastre of the extinct village of Libkovice, Most district. The site is located in lowland near the former confluence of the Lomský and Radčický streams (50.57479°N, 13.68595°E). In an irregularly oval grave pit (grave 233/18), the skeleton of an individual lying on the left side was found, identified archaeologically as a woman. An amber necklace containing at least 20 corals of various shapes and sizes was found around the neck of the deceased. Another necklace of several dozen large drilled animal teeth was hung in the rib cage area. In front of the body there was a belt consisting of at least fifteen rows of several hundred drilled animal teeth of various types and sizes [2]. One of the drilled dog teeth (*caninus inferior dexter*) was chosen as material for ancient DNA analysis in this study.

Mikulovice, Pardubice district, east Bohemia, Czech Republic

Contact person: Michal Ernée

The site is located in Pardubice district, east Bohemia, Czech Republic (49.99200°N, 15.77686°E) and was excavated during rescue excavation in 2006–2012 (J. Frolík, R. Sedláček). The site contains thousands of sunken settlement features, about 100 Early Bronze Age (EBA) graves, and three settlement features containing 109 skeletons were documented in several groupings. With exception of two Proto-Únětice graves (No. 95 and 96), the graves mostly belong to the Classic and Post-Classic stages of the Únětice culture. The inhumation burials are very rich in so-called “exotics”, especially amber artefacts, which are present in 28 graves. The EBA cemetery has been completely analysed and published as a monograph [11] that is available open access on AcademiaEdu https://www.academia.edu/43585678/Mikulovice_Early_Bronze_Age_Cemetery_on_the_Amber_Road). It contains the complete anthropological (P. Stránská, E. Zazvonilová), palaeopathological (L. Vargová, K. Vymazalová, L. Horáčková), demographical, anthropomorphic (epigenetics; P. Velemínský, J. Cvrček) and osteological (R. Kyselý) analyses [11].

The two samples included in this study came from grave 42 (feature 2040). This grave contained the skeleton of a child (6,5–7,5 years): right-sided crouched burial, head towards the south, east-faced, with palaeopathological lesions suggestive of enamel hypoplasia, cribra orbitalia sin. aDNA screening identified plague [10]. Grave goods: small bronze chisel behind the head, simple bronze armring on the left-hand wrist, three amber beads, one drilled dog tooth by the waist, and animal bone (pig scapula [12]) in front of the knees. Archaeological dating: Classic Únětice culture. Radiocarbon dating: MAMS-30479 (3550±19) 1950–1779 BCE cal 2-sigma. [11]. Pandora No.: MIB054. NM Prague Inv. No.: P7A 43141.

Two samples, one drilled dog canine tooth (context 5031) and a pig scapula (context 5032, fragmented postmortem), both offerings from grave 42, were selected for this study.

Mochov, Czech Republic

The excavation of site Mochov 3 (location „Na Nehvizdku“; 50.13336°N, 14.77475°E) was carried out in 1980 by the Museum of Čelákovice city (J. Špaček). The sunken feature no. 17 was dated archaeologically to Salzműnde phase of Funnelbeaker Culture by M. Zápotocký (unpublished). This dating was later confirmed by radiocarbon analysis of the sample from the dog skeleton (4669 ± 24 BP, 3519 – 3369 cal BCE (95%); analysis CRL-20_168: unpublished data). A dog skeleton found in the feature together with a child skeleton represents a non-standard finding undoubtedly with ritual connotation, despite the nature of deposition of the dog body is not clear from the terrain documentation. One tooth (*premolar 4 inferior dexter*) from the dog skeleton was chosen for DNA analysis.

Prague-Březiněves, Czech Republic

Several graves of Corded Ware Culture (CWC) were excavated in Prague-Březiněves in 2006 (location Na Boleslavce; 50.17039°N, 14.48964°E) by ARCHAIA Praha z.ú. (K. Svoboda). The excavation represents a typical CWC cemetery located in the lowlands of the central part of the Bohemian distribution of the CWC. The left-sided burial no. 10 contained an extraordinarily large number of drilled teeth and shell beads (probably the largest within the whole archaeological culture) arranged in strips (probably fixed to clothing). The finds represent at least 412 whole teeth from at least 78 dogs, and 4000-5000 shell beads, including two large solar discs of *Margaritifera auricularia* [1,2]. Two dog teeth from this collection, specifically 2x *incisivus 3 superior dexter*, were analysed here. The same anatomical position guarantees that the teeth originate from two different dog individuals.

Germany

Bürgermeister-Ulrich-Straße 100, Augsburg, Germany

The site is located in the south of today's city of Augsburg, Bavaria (48.32351°N, 10.89306°E) just a few hundred meters south of the Bell Beaker cemetery of “Augsburg – Universitäts-Sportgelände”. Large scale archaeological rescue excavations were carried out in 1986 until 2002 within the industrial park leading to the discovery of Early Bronze Age longhouses and several probably contemporaneous pits containing ceramic materials as well as animal bones, which are part of this study. Alongside these settlement evidences, undated ring ditches and Late Neolithic burials were discovered. The oldest burials are two graves situated next to each other attributed to the CWC. In total, twelve Bell Beaker burials were found distributed in three separate groups. Graves 1–8 were found in the northwest of the excavation site, graves 9–11 were further south next to the CWC burials, but separated by approximately 10-15 meters, and grave 12 was found around 120 meters northeast of them. The latter was constructed with a ring ditch. From the small faunal assemblage,

two specimens were selected for ancient DNA sampling: This includes a thoracic vertebra of a small domestic ruminant and a cattle metacarpal.

Gewerbegebiet Nord, Kleinaitingen, Germany

The site of Kleinaitingen – Gewerbegebiet Nord, Bavaria (48.22281°N, 10.84499°E) is located ca. 16 km south of today's city of Augsburg on the upper gravel terrace of the Lech River. Apart from some prehistoric (mostly Late Bronze Age settlement) features, a large Early Bronze Age cemetery was documented between 2012 and 2014 by members of the local history and archaeology society (Arbeitskreis Augsburg Süd). It was almost entirely excavated; only the southern limit is still covered by a modern pathway. In total 63 graves with 72 individuals were documented, most of them inhumations, making it one of the largest Early Bronze Age cemeteries in southern Germany [13]. All individuals/inhumations were buried in a crouched position with typical gender-specific orientation and body placement of the Bell Beaker and Early Bronze Age type. The radiocarbon dates span from 2116 until 1514 cal BCE, with the majority of the dates falling between 1900 and 1700 cal BCE. Animal bones were a part of the grave-good assemblages. For this study animal bones from four graves (no. 30, 45, 52 and 62) as well as two pieces from a contemporary ring ditch (feat. 13) were sampled. Due to the burial rites characteristic for the Early Bronze Age in the northern Alpine foreland, the faunal record in grave contexts is largely limited to teeth and mandibles. Thus, sampling focused on these skeletal elements. Five cattle teeth and one cattle mandible representing individuals of different slaughter age stages were selected for this study.

Obere Kreuzstraße, Königsbrunn, Germany

The site of Königsbrunn – Obere Kreuzstraße is located ca. 10 km south of today's city of Augsburg, Bavaria (48.26656°N, 10.87857°E) on the upper gravel terrace of the Lech River at the western edge of the town of Königsbrunn. It was excavated during pre-construction works in 2007 in its full extent. All 48 grave pits of the Early Bronze Age cemetery were oriented NNE-SSW or shifted slightly clockwise and comprised five distinct groups of graves [13]. In total 50 inhumations could be documented with individuals buried in a crouched position typical of Bell Beaker and Early Bronze Age gender-specific body positioning. The radiocarbon dates range between 2136 and 1780 cal BCE. Most of the dates fall between 2040 and 1880 cal BCE. Animal bones, mostly cattle teeth, were a regular part of the grave good assemblages. For this study six upper or lower cattle molars (one with an alveolar fragment attached) from different graves as well as a mandible and teeth from a contemporary deposition of a young cattle within the northern grave group (feat. 215) were sampled.

Postillionstraße, Haunstetten, Germany

The site of Haunstetten - Postillionstraße is located ca. 7,5 km south of today's city centre of Augsburg, Bavaria (48.29573°N, 10.89103°E) on the upper gravel terrace of the Lech River in the

southern part of the district Haunstetten. The Early Bronze Age cemetery was excavated in 1992 by the city archaeology of Augsburg before housing estates were built. Parts of the site could not be documented, but large areas of the Early Bronze Age cemetery were excavated. Apart from the cemetery, a small ditch and a horse burial from the Roman period were found. The Early Bronze Age cemetery contained at least 40 graves with 41 individuals [13]. All individuals were inhumations and buried in accordance with the gender-specific orientation and body positioning of the Bell Beaker and Early Bronze Age type. Five graves (feat. 44, 50, 65, 137 and 111) were accompanied by long post alignments, making them outstanding burial monuments/individuals. Radiocarbon dates gathered from skeletal remains range between 2197 and 1772 cal BCE. Most of the dates fall between 2140 and 1880 cal BCE. Most of the animal remains sampled in this study stem from postholes of these alignments. Additionally, one sampled tooth comes from a ring ditch (feat. 49) surrounding an Early Bronze Age burials (feat. 50), another tooth was part of the grave good assemblage of one of the burials (feat. 99). The faunal assemblage exclusively consists of cattle remains, of which five molars and two mandibles have been included in this study.

Universitätsstraße, Augsburg, Germany

The site of Augsburg - Universitätsstraße is located in the middle of the university campus in the south of today's city of Augsburg, Bavaria (48.33262°N, 10.89402°E). Besides various prehistoric archaeological features, a large pit with a substantial amount of Early Bronze Age pottery accompanied by animal bones was excavated (feat. 43). The pottery can be dated roughly around 1800-1600 BCE. The faunal assemblage consists of only seven identified specimens, representing the livestock species characteristic for the Early Bronze Age economy of the Northern Alpine foreland: cattle (n=1), sheep (n=1), sheep/goat (n=2), and pig (n=3). From this assemblage, we selected a Phalanx proximalis (cattle), a Phalanx medialis (sheep) and a scapula (pig) for sampling.

Unterer Talweg 85, Haunstetten, Germany

The site of Haunstetten – Unterer Talweg 85 (today street no. 49) is located approximately 4.5 km south of today's city centre of Augsburg, Bavaria (48.31966°N, 10.89153°E). This large-scale excavation took place in 1999 and uncovered Early and Late Bronze Age settlement remains as well as burials with inhumations from the Bell Beaker Period and Early Bronze Age [13]. The Bell Beaker graves have been found in two concentrations, ca. 170 metres apart from each other. The northern group contained five single burials oriented N-S. The radiocarbon dates range from 2465–2143 cal BCE. The two single graves of the southern group could be contemporaneous to the burials of the northern group (2456–2141 cal BCE). Almost in the middle of both Bell Beaker burial groups, but ca. 70 metres to the east, a small part of a presumably larger Early Bronze Age cemetery was unearthed with radiocarbon dates spanning between 2025-1885 cal BC. West of these Late Neolithic and Early Bronze Age burials, remains of a large Early Bronze Age settlement were uncovered with at least seven longhouses accompanied by smaller storage buildings. On the western side of the excavated area, at least two Late Bronze Age timber buildings were identified as well as several La Tène Period (Late Iron Age) pits with human remains. Faunal remains include a total of 24 identified specimens,

distributed among cattle (n=12), sheep (n=1), sheep or goat (n=2), pig (n=7), red deer (n=1) and beaver (n=1). Of these, 18 have been selected for sampling (several with multiple subsamples).

Unterer Talweg 111, Haunstetten, Germany

The site of Haunstetten – Unterer Talweg 111 is located ca. 5 km south of today's city centre of Augsburg, Bavaria (48.31421°N, 10.89007°E). Four neighbouring plots were archaeologically investigated due to the construction of three supermarkets, among other things (Unterer Talweg 109-115). In the northern area, a circular ditch and several Late Bronze Age cremation burials came to light. The cremation graves extended as far as the plot at Unterer Talweg 113. At least seven Early Bronze Age buildings were uncovered in the area of Unterer Talweg 111, 113 and 115. Ten Early Bronze Age burials were found to the north-east of the settlement (Unterer Talweg 111) which can be dated to the middle phases of the Early Bronze Age (ca. 2050-1800 BC) [13]. The sampled animal bone material comprises a mandible and a tooth row with alveolar bone, both from sheep.

Moldova

Petreni, Moldova

The settlement of Petreni (47.91704°N, 27.87686°E) is located on a hill plateau within the hilly landscape of the Bălţi Steppe. Belonging to the Cucuteni-Tripolye-Complex, where settlements, especially in the first half of the 4th Millennium BCE, show the tendency to form huge agglomerations of houses, which occupy very large spaces of up to 320 ha. The houses were built and appear to have been deliberately burnt within a rather short period of less than 200–300 years. Well-preserved zooarchaeological remains were recovered during the excavations led by Dr R. Uhl. A total of 3146 bones were identified and taxonomically assigned by N. Benecke and M. Hochmuth (unpublished). Cattle were the predominant species (Number of identified specimens (NISP)=1515) while sheep/goat and pigs represented 34% and 10.6% respectively of the identified material. For study, 30 teeth were selected from secure contexts by R. Uhl, R.E. Gillis and A.K.W. Runge.

Poland

Giecz 10, Poland

This site is located in central Poland in the Wielkopolska region (52.32083°N, 17.37361°E) and was discovered by Józef Kostrzewski in 1928 [14,15]. The first excavation was carried out in 1948 by Bogdan Kostrzewski [16]. Giecz was one of the most important centers in the formation of the Polish state during the early Middle Ages. Historical records and particularly archaeological research indicate that there was a stronghold of great political, economic, and military importance located at Giecz from the second half of the ninth century to the thirteenth or fourteenth century. Numerous settlements and cemeteries were located around this stronghold. In recent years, archaeological research at Site 10 has revealed one of these settlements. Materials collected from numerous contexts, including

pottery, clay, metal, and stone artefacts, as well as jewelry and bones of both humans and animals, are sources of data on the chronology, demography, and everyday life of the site, and can be employed as indicators of formational and post-depositional processes. In this way we know, mainly from the study of pottery, that the settlement at Site 10 existed between the ninth and tenth centuries, and was therefore contemporaneous with the oldest pre-state development phase of the Giecz stronghold—which highlights its importance in research. A total of 62,466 animal bones were obtained from the archaeological contexts of Site 10 during archaeological investigations in 2014–2019 and 2021–2023, some of which have undergone zooarchaeological and taphonomic studies by K. Pawłowska [17–20]. Overall, the proportion of specimens with pathological changes did not exceed 4% [19–22]. Changes such as arthropathies, inflammatory diseases, diseases connected to the environment, dental anomalies, oral pathology, and traumatic lesions were found. In this study, 15 samples of different skeletal elements from cattle, pigs, sheep and/or goats, and red deer were selected for DNA analysis by K. Pawłowska (**Supplementary Data 1** and **Supplementary Data 2**)

Zdrojówka, Poland

This archaeological site is located in central Poland in the Kujawy region (52.43778°N, 18.74722°E). It was discovered in 1957, when a human grave and a neighboring double burial of cattle, belonging to the Globular Amphora Culture, was uncovered [23,24]. The west-to-east oriented grave was surrounded by stones and clay. Blades were reported from this context, as well as other artifacts such as amphorae [23,25]. The burial includes examples of ritual patterns of animal deposits equipped with pots [26]. Among the specimens, a mandible was selected for study: this specimen does not show evidence of pathology.

Gdańsk, Św. Wojciecha site, Poland

This site is located in the city of Gdańsk in Pomerania in the northern part of Poland (54.37069°N, 18.61298°E). Archaeological research was conducted here in the 1950s. The archaeological contexts of the site have been dated to the ninth to thirteenth centuries [27]. The animal remains were studied by Kubasiewicz [28], though specimen pathology was not then reported. However, a palaeopathological study was carried out by K. Pawłowska in 2021. Among the specimens with pathological changes, a first phalanx of cattle was selected for study: this specimen shows a lytic lesion within the shaft.

Site 63, Krzczonowice, Poland

Archaeological site no. 63 at Krzczonowice (50.86582°N, 21.49391°E), in Świętokrzyskie province, Poland, is a part of the larger excavation area within the Archaeological Museum and Krzemionki Reserve (UNESCO), which was initiated in the 1930s [29–31]. The explorations at Krzczonowice, which began in 2004, are located in the middle and lower basin of the Kamienna river in the northern part of the Sandomierz Upland. Indirect chronology has dated this settlement to the Globular Amphora Culture. Direct radiocarbon dating has shown that a bone fragment from the site comes from 2476–2293 cal BC (4425–4242 cal BP). Special attention was paid to object no. 33, which was

identified as a sacrificial pit with an almost complete bovine skeleton deposit. The oval pit is circa 180 cm deep and contains the skeletal remains in partly anatomical order [31]. The total number of bone fragments was 302 and species identification was possible in the case of 78.15% items (NISP = 236). Over 85% of the NISP was skeletal material coming from a taurine cattle individual. Other species included pigs, sheep/goats, horses, lynx, and humans. Before deposition, the cattle cadaver was probably partitioned. The thoracic, lumbar, sacral, and caudal part of the vertebral column, together with the right pelvic limb, was unearthed as articulated laying on the left flank. The position of the cervical vertebra and the mandible suggest that the head and neck were bent caudally and positioned on the right side of the animal trunk. The skull was unhorned. The bones of the left pelvis and of both thoracic limbs were not in anatomical position. The only visible pathological feature was noted within the greater trochanter of the right femur, as a massive bone tissue loss process that led to the formation of a concave area (cortical and spongy bone destruction and lytic process). Further palaeopathological analysis may be able to identify the potential cause of the disease. Genetic analysis was carried out using bone material from the lesion location.

Site 154, Inowrocław, Poland

This site is located in the Kujawy region in Poland (52.79886°N, 18.26387°E). The archaeological rescue excavation at Site 154, Inowrocław, was conducted in 2009 by Marcin Woźniak. The stratigraphy of the site, along with the archaeological context, was established using ceramics found to be from the Bronze Age (VEB–HaC: 8th-7th century BC). During the excavation, animal remains were recovered from the settlement complex, of which some assemblages were studied [32]. Pathological lesions were found on twenty specimens. In the present study, 17 samples representing a range of skeletal elements were selected for DNA analysis, as they showed inflammatory diseases (**Supplementary Data 2**). These specimens represent horses, aurochs, cattle, pigs, sheep or goats, and dogs.

Moszna Wieś, Poland

The site is located in the Mazowsze region in northeast Poland (52.18091°N, 20.74727°E). Archaeological research was conducted at the site, leading to the discovery of a settlement with features of the Jastorf culture; dating to the younger pre-Roman period in Moszna Wieś was based solely on the analysis of ceramic material. Animal bones were also recovered during this work (NISP = 255). The assemblage was studied taxonomically, demographically, and taphonomically [33] and three specimens with pathological changes were found. Two of these, both mammalian long bones, were selected for the DNA analysis, as they showed inflammatory diseases (**Supplementary Data 2**). Their state of preservation did not allow for species determination, but it was possible to assign them to the cattle size class.

Izdebno Kościelne, Poland

This site is located in the Mazowsze region in northeast Poland (52.13764°N, 20.52185°E). The site was discovered in 1971 by Woyda, and archaeological research was conducted in 1976–1978 by

Górna and later Nowakowski, and then again in 2008-2009 by Rozen and later Piłatowski. The archaeological contexts and recovered materials were dated to the Palaeolithic, Neolithic, early Bronze Age, early Iron Age, pre-Roman period, Roman period, and early phase of the migration period, as well as late medieval and modern times [34]. Archaeological structures were uncovered and bulk materials were obtained, including more than 6000 animal bones. These collections have been investigated in terms of taxonomy, demographics, and taphonomy [35]. In total, 36 specimens with pathological changes were found and two samples were selected for DNA analysis. (**Supplementary Data 2**). Both samples originated from cattle and belonged to the contexts dated to older and younger pre-Roman period and the late Roman period, respectively.

Romania

Pietrele-Măgura Gorgana (Pietrele), Romania

Today the settlement mound Măgura Gorgana is situated at a distance of 7 km to the Danube river in Romania (44.06887°N, 26.15900°E). In the 5th millennium it was situated directly at a lake through which the Danube flowed. It was probably a so-called ria which was open to the Black Sea [36]. The 11 m high settlement mound provides a continuous stratigraphy from 4600 to 4250 BCE which is connected with the Gumelniţa culture of the South Romanian Early Copper Age. From around 4500 BCE a flat settlement was erected around the settlement mound. There were certain social differences between the settlers on the mound and those around the mound [37]. The main focus of the economy was probably fishing [38]. Other animal remains were studied by N. Benecke [39–41]. The Earlier Gumelniţa layers (4600-4450BCE) in Măgura Gorgana domestic animals are represented primarily by cattle (70%), followed by pigs (15%) and small ruminants (sheep and goats; 15%). The proportion of wild boar and red deer did not exceed 8%. In contrast, husbandry from the subsequent Gumelniţa settlement (4450-4250BCE) attests to husbandry mainly of pigs (56%), followed by cattle (33%) and sheep and goats (11%) [39]. Remains of wild animals like aurochs, wild boar, red deer, and wolves are significantly higher. K. Pawłowska conducted a study in 2022–2023 to select specimens with pathological changes for this study. 83 skeletal elements (AZP-102–AZP-184) were selected for DNA analysis; these showed inflammatory diseases, dental anomalies, oral pathology, and inflammation associated with arthropathy. These specimens represent cattle, pigs, sheep, goat, sheep or goats, dogs, aurochs, wild pig, red deer and beaver (**Supplementary Data 2**).

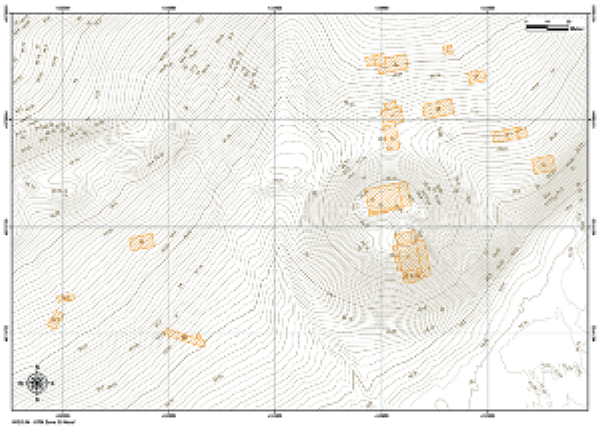

Image (CC BY SA 4.0 license) of the Pietrele site, with the tell and its immediate surroundings and excavation areas marked (D. Nowacki from Benecke et al., [39]).

Russia

Ipatovo 3, North Caucasus, Russia

The so-called 'Great Barrow', kurgan 2, of Ipatovo 3 is one of the most important Bronze Age sites in the steppes north of the Caucasus. It is located on the outskirts of the city of Ipatovo, about 120 km northwest of Stavropol, Russia (45.67661°N, 42.91509 E). The mound is one of three larger mounds and part of a long line of smaller ones. It is situated in the valley of the Kalaus River, which rises on the northern slopes of the Caucasus Mountains and flows into the Manych River to the north that is an important north-south connection on the eastern edge of the Stavropol Upland between the Caucasus mountains and the steppes.

Kurgan 2 was one of the largest burial mounds in the area, measuring 7 m in height and over 100 m in diameter, and contained three different phases of Bronze Age occupation and a later reuse during the Sarmatian epoch. The mound was excavated in 1998/1999 over a period of one and a half years as part of a rescue excavation by the heritage organization 'Nasledie' in Stavropol, directed by A. B. Belinskiy [42]. The Bronze Age graves are part of bioarchaeological studies including isotopic analysis, palaeogenetics and palaeoproteomics [43–47]. The ribs and vertebrae from one serpent excavated at the site were analysed for DNA.

Komsomolec 1-Marfa, North Caucasus, Russia

The huge burial mound Komsomolec 1-Marfa was a single standing mound on the right river terrace of the Zolka creek, located 7,2 km west of the north-western outskirts of the city of Novopalovsk, Stavropol region, Russia (43.97728°N, 43.51637 E). Today, the area is a piedmont steppe environment but was probably more densely forested in the past. The watershed along the river is covered with former barrows, now chiefly ploughed out. The site was excavated in a joint expedition of the local heritage organization 'Nasledie' and the Eurasia department of the German Archaeological institute, directed by A.B. Belinskiy and A. Nagler. Beside the mound a circular ditch enclosure is visible on aerial and satellite images, as well as in a magnetometry survey conducted in 2009 and 2011. The mounds height was 8,5 m, it had an oval shape, the diameter was 62 m along the west-east axis and 52 m along the north-south axis. The mound was built in several consecutive years and altogether 69 graves have been excavated in the mound. The initial mound dates to the Early Bronze Age, the local late Maykop epoch, yet the central grave was destroyed. Nine more graves have been dated Maykop, while subsequent graves have been excavated as well: 48 date to different cultures of the Middle Bronze Age, seven are of Late Bronze/Early Iron Age date, one is Sarmatian, and three modern individuals were found. Beside the burials, a ditch with horse skeletons was opened, probably dating to Sarmatian times. Additionally, ritual areas with human skeletons were discovered beside the mound, dating to the Early Iron Age and the medieval period.

The site is prepared for a full publication and is central in bioarchaeological studies including isotopic analysis, palaeogenetics and palaeoproteomics [43,46,47].

One sample, the incisor of a caprid, was included in this study. It originated from Grave 53, a single inhumation of the Catacomb culture (2800-2600 cal BC). The human skeleton was found in a catacomb grave lying in a crouched position on the left side. The floor was sprinkled with chalk and below the individual organic brown matter was found. Grave gifts included a bronze knife, two vessels, and animal bones.

Marinskaya 5, North Caucasus, Russia

Marinskaya 5 was another huge burial mound, located near the village Marinskaya, Stavropol region, Russia (43.92500°N, 43.53000 E). The single-standing burial mound was excavated in 2009 by a team of the Lomonosov Moscow State University, the Institute of Archaeology RAS and the heritage organization 'Nasledie', directed by A.R. Kantorovich and V.E. Maslov. The kurgan is situated on the high terrace of the river Kura. The mound was slightly oval with a diameter of 34 to 40 m and 4.3 m high. At a distance of 10-16 m a ditch of 1,5 m depth surrounded the mound, which was detected on aerial images and excavated later. In the central part of the mound, the excavations uncovered three mound-shells phases dating to the Early Bronze Age Maykop epoch and a fourth one that was constructed during the Middle Bronze Age. A sequence of six burials dating to the Maykop period, while 17 Middle Bronze Age graves are associated with the local North Caucasian culture, one is a Catacomb culture grave, and three date to the final Middle Bronze Age. In the Sarmatian period six graves and several ritual complexes were added to the older mound.

In several graves, among them late Maykop grave 25 and the North Caucasus graves 19, 23, 30/30a, paired cattle skulls were found. They place this site among one of the earliest where the use of cattle as draught animals is documented in a chronological sequence. One cattle premolar was selected for DNA analysis.

The site is partly published and central to a multidisciplinary study including stable isotope analysis, physical anthropology and palaeogenetic investigations [43,46–49]

Ransyrt 1, North Caucasus, Russia

Ransyrt-1 is a wall system consisting of four semicircular ramparts with a diameter of about 300 m near the modern city of Kislovodsk in the region of the Caucasian Mineral Waters, Russia (43.84157°N, 42.30286 E). The site is located at an altitude of about 1,800 m above sea level on the edge of a high plateau above the Podkumok River, at one of the most important pass roads in the central part of the North Caucasus. Three semicircular ramparts enclose a central area, which is itself surrounded by a wall. A magnetometer survey indicated intensive use of the site. Joint excavations in 2015 by heritage organization 'Nasledie' and the Eurasia department of the German Archaeological institute, directed by A.B. Belinskiy and S. Reinhold revealed stone structures and sunken rooms, for which the finds and features suggest ritual use [50]. All features date to the start of the Late Bronze Age (1850-1600/1500 cal BCE).

Four main phases of use were identified and dated, the first of which is the active use of the structures and during which an exceptionally large amount of animal bones and ceramics were left at the site. Presumably, they are the remains of ritual feasts or sacrifices. An analysis of the animal bones supports the thesis of selective, ritual use and suggests that those consumed in Ransyrt 1 were not kept in the surrounding area but came to the settlement from outside [51].

The cow tooth, AZP-013, included in this study comes from the Late Bronze Age stock of animal bones, while the souslik mandibula, AZP-014, was excavated from the Late Bronze Age layers but might equally be a later intrusion.

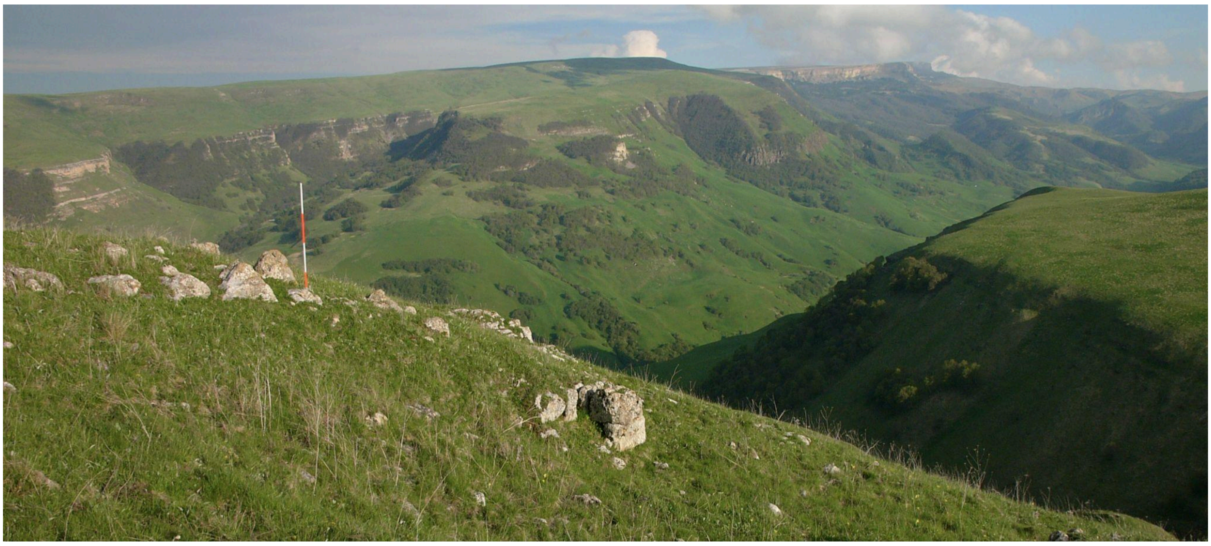

View from the ritual site Ransyrt 1 overlooking the Podkumok valley and the mt. El'brus Massiv (5642 m asl). Photo: Sabine Reinhold.

Ransyrt 2, North Caucasus, Russia

The second site, Ransyrt 2 (43.84176°N, 42.29742 E), is about 200-300 m west of the outermost rampart of Ransyrt 1. The site consists of a linear cluster of small stone socketed. Surface collections revealed Late Bronze Age material, but no excavations have been carried out so far.

A rodent skull was selected for DNA analysis. It comes from surface collections and may stem from the Bronze Age cultural layers, but could also be a modern intrusion.

Sharakhalsun 3, North Caucasus, Russia

Sharakhalsun 3 is an archaeological site in the Manych Steppe, about 50 km southeast of the town of Divnoe, Stavropol region, Russia (45.72094°N, 43.98044 E). It is one of several long rows of burial mounds running parallel to the Kalaus River. Many similar rows of burial mounds are located nearby, forming a unique burial landscape.

In 2001, 12 barrows were documented and five of them excavated in the course of rescue excavations by the heritage organization 'Nasledie', directed by A. V. Yakovloev.

Mound 1, from which the studied dog tooth originated, dates to the Middle Bronze Ages' late Catacomb Culture (2400-2200 cal. BCE). The tooth itself was discovered in the mound fill and can either date to the Bronze Age or is a later intrusion.

Sharakhalsun 6, North Caucasus, Russia

Sharakhalsun 6 is another line of burial mounds in the Manych Steppe, about 50 km southeast of the town of Divnoe, Stavropol region, Russia (45.72094°N, 43.98044 E). It runs parallel to the Sharakhalsun 3 site and equally to the Kalaus River. In 2001, during rescue excavations, 16 mounds were excavated by the heritage organization 'Nasledie', directed by A. V. Yakovloev. It is part of a bioarchaeological study including stable isotope analysis and palaeogenetics [43,47].

The cattle tooth included here came from mound 3, which is dated to the late Catacomb Culture (2400-2200 cal. BCE). The animal bones were found in a ritual context in the mound shell. The cattle tooth came from 'animal 1' in the archaeological documentation.

Stoderovskoe 1, North Caucasus, Russia

The site of Stoderovskoe 1 (43.80154°N, 44.85261 E) is a settlement dating to the Early Bronze Age Maykop epoch. It is located near the village Stoderovskaya in the Stavropol region, Russia, about 13 km east of the city of Mozdok. The site is located in the valley of the Terek river, on its left bank.

During rescue excavations in neighbouring burial mounds in 2008 several pits with large amounts of pottery and animal bones, and animal bone assemblages were excavated by an expedition of the heritage organization 'Nasledie' under the license of A. V. Lychagin. Two molars, one from cattle and one from pig, were selected for DNA analysis. They stem from one of the pits and have also been included in a stable isotope analysis study, which is in preparation for publication.

Tomatniy 1, North Caucasus, Russia

The burial mounds of Tomatniy 1 (44.04619°N, 42.77540 E) are located in the vicinity of the city of Pyatigorsk, Stavropol region, Russia. Originally excavated by the heritage organization 'Nasledie', animal bones from the Bronze Age mound 1, grave 8 were collected for a stable isotope study. From these, one tooth from an adult fox was selected for DNA analysis.

Turkmenistan

Monjukli Depe, Turkmenistan

The small mound of Monjukli Depe in the foothills of the Kopet Dag in south Turkmenistan (36.84833°N, 60.41778°E) was occupied in the sixth millennium BCE during the Neolithic Jeitun period and, after a long hiatus, reoccupied in the Early Aeneolithic (Chalcolithic) in the fifth millennium BCE. Initially discovered and excavated in Soviet times [52], fieldwork at the site was resumed from 2010 to 2014 by a team from the Freie Universität Berlin. The Neolithic levels excavated in only small proportions were characterized by diffuse layers with only little architecture, containing variable densities of artifacts. The extensively investigated Aeneolithic levels exposed substantial portions of superimposed and well-preserved mudbrick architecture [53,54]. The excavations at Monjukli Depe have uncovered an extensive collection of approximately 53,000 animal bones. The taxonomic identification of the material was conducted by M. Hochmuth and N. Benecke. They consist predominantly of remains from domesticated caprines (>90%) both in the Neolithic and Aeneolithic assemblages, with sheep outnumbering goats [55–58]. A markedly smaller number of domesticated cattle were recovered. Dog bones complete the spectrum of domesticated animal taxa. The inhabitants of Monjukli Depe focused almost exclusively on the rearing of small ruminants. Wild fauna accounts for less than 5% of the identified remains, with species including onager, gazelle, wild sheep, and foxes, indicating that hunting played a very minor economic role for the inhabitants. Multi-isotope analysis of tooth enamel (carbon, oxygen, strontium) and bone collagen (carbon, nitrogen) suggests substantial dietary variability among caprines, with evidence of C_4 plants contributing to the diet of part of the herd on both seasonal and long-term scales [58,59]. This indicates herding practices that utilized the micro-variability of the local landscape, with sheep and goats kept in small flocks across ecologically diverse areas close to the settlement.

Nine sheep/goat teeth originating from features of the Aeneolithic occupation were selected for DNA analysis.

Uzbekistan

Tilla Bulak, Uzbekistan

The Tilla Bulat site is situated near the border tripoint of Uzbekistan, Turkmenistan, and Afghanistan, in ancient Bactria (37.71105°N, 66.80059°E), about 850m above sea level. The settlement developed in the early phase of the Late Bronze Age, which correlates with the Sapalli Culture—that is, a local variant of the Southern–Central Asian Namazga Cultures. The site extends over an area of about 0.4 ha and is dated to the twentieth to nineteenth centuries BC. Exploration in this area has been carried out by the Institute of Near Eastern Archaeology, Ludwig Maximilian University of Munich, by the Tocharistan expedition of the National Institute of Fine Arts in Tashkent, and by the University of Termez. The excavations on site were conducted from 2007 to 2010, uncovering almost 40% of the examined settlement area [60,61]. Animal bones (NISP = 10,869) were recovered from the archaeological contexts of the two main phases of the site. Sheep and goats dominate the assemblage (92%), with cattle representing 5% of the assemblage, while the proportion of pigs was negligible [62]. The preliminary analysis had recorded no pathologies. Palaeopathological analyses

conducted by K. Pawłowska for this study recorded a high frequency of pathologies, particularly within sheep/goat remains. Of these, 105 skeletal elements were selected for DNA analysis. They showed evidence of inflammatory diseases, dental anomalies, oral pathology, inflammation associated with arthropathy, and traumatic lesions. These specimens represent horses, cattle, sheep, goat, sheep/goats, dogs, fox and gazelle (**Supplementary Data 2**). A fragment of a cattle rib (AZP-285) displays a lesion that could be indicative of a neoplastic, tumorous, or inflammatory disease such as osteolytic cancer or tuberculosis.

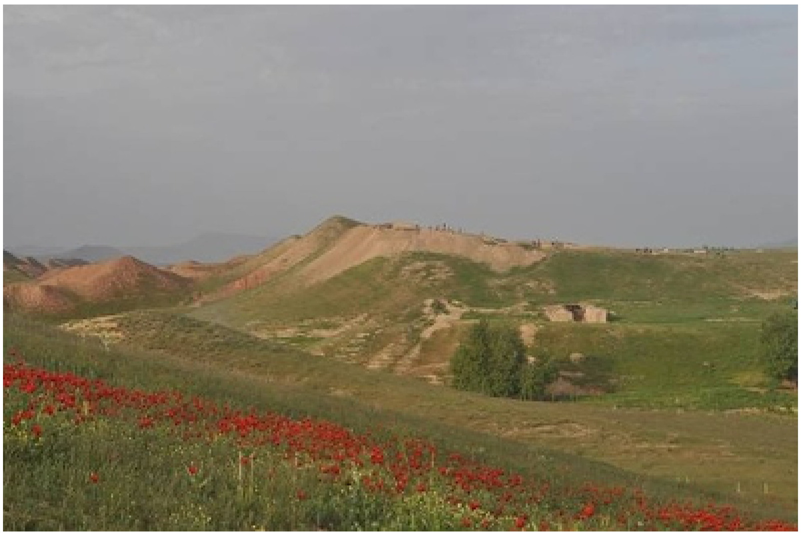

Photo of the Tilla Bulak site (by K. Kaniuth).

References

1. Kyselý, R., Dobeš, M. & Svoboda, K. Drilled teeth and shell artefacts from a grave at Prague-Březiněves and a review of decorative artefacts made from animal material from Corded Ware culture in the Czech Republic. *Archaeol. Anthropol. Sci.* **11**, 87–131 (2017).
2. Kyselý, R. Dog size and variability in the Late Eneolithic: Drilled teeth from Corded Ware graves in Bohemia. *Int. J. Osteoarchaeol.* **31**, 487–505 (2021).
3. Dobeš, M. & Zápotocký, M. Pozdní fáze kultury nálevkovitých pohárů v severozápadních Čechách: sídliště Brozany nad Ohří. *Archeol. rozhl.* **55**, 451–503 (2013).
4. Kyselý, R. An analysis of osteological material from the late Funnel Beaker culture settlement in Brozany, northwestern Bohemia. *Archeol. rozhl.* **65**, 504–534 (2013).
5. Daněček, D. & Klementová, J. Hostivice, k. ú. Hostivice, výstavba logistického centra Tulipán Park

(př. č. 1/2007). Archeologické výzkumy Středočeského muzea v Roztokách u Prahy v roce 2007. *Středočeský vlastivědný sborník* **26**, 104–105 (2008).

6. Daněček, D. & Smíšek, K. Hostivice, k. ú. Hostivice (př. č. 20/2010). Středočeské muzeum v Roztokách u Prahy. Archeologické výzkumy v roce 2010. *Středočeský vlastivědný sborník* **29**, 170–172 (2011).
7. Daněček, D., Klementová, J., Kvěchová, E. & Nový, P. Hostivice, okres Praha-západ. Středočeské muzeum v Roztokách u Prahy. *Středočeský vlastivědný sborník* **32**, 88–91 (2014).
8. Nicolas, C. Bracer Ornaments! An investigation of Bell Beaker stone 'wrist-guards' from Central Europe. *JNA* 15–107 (2020) doi:10.12766/JNA.2020.2.
9. Papac, L. *et al.* Dynamic changes in genomic and social structures in third millennium BCE central Europe. *Sci Adv* **7**, (2021).
10. Andrades Valtueña, A. *et al.* Stone Age Yersinia pestis genomes shed light on the early evolution, diversity, and ecology of plague. *Proc. Natl. Acad. Sci. U. S. A.* **119**, e2116722119 (2022).
11. Ernée, M. *et al. Mikulovice. Pohřebiště starší doby bronzové na Jantarové stezce – Mikulovice. Early Bronze Age Cemetery on the Amber Road*. (Památky archeologické, Supplementum 21, Praha, 2020).
12. Kyselý, R., Limburský, P., Šumberová, R., Langová, M. & Ernée, M. Scapulae and phalanges as grave goods: a mystery from the Early Bronze Age. *Archaeol. Anthropol. Sci.* **12**, 1–34 (2020).
13. Massy, K. *Gräber der Frühbronzezeit im südlichen Bayern. Untersuchungen zu den Bestattungs- und Beigabensitten sowie gräberfeldimmanenten Strukturen. Mit Beiträgen von Nadja Hoke, Anja Staskiewicz, Wolf-Rüdiger Teegen und Stephanie Panzer*. (Verlag Michael Laßleben, Kallmünz, 2018).
14. Jażdżewski, K. & Rajewski, Z. Sprawozdanie z wycieczek Akademickich Koła Polskiego Towarzystwa Prehistorycznego za rok akademicki 1927/28. *Z Otchłani Wieków* **3**, 67–68 (1928).
15. Miciak, M. & Agnew, A. Cmentarzysko wczesnośredniowieczne w Gieczu, stan. 10, woj. Wielkopolskie- wyniki badań z lat 2014–2019 [An early medieval cemetery at site 10 in Giecz,

Wielkopolska province: Research results from 2014–2019]. *Studia Lednickie* **20**, 125–195 (2021).

16. Kostrzewski, B. Gród w Gieczu w świetle źródeł pisanych i wykopalisk. *Z Otchłani Wieków* **20 (9–10)**, 149–154 (1951).
17. Pawłowska, K. Przemiany gospodarcze we wczesnośredniowiecznej osadzie w Gieczu [Economic changes in the early medieval settlement of Giecz]. *Museum manuscript of zooarchaeological study,* 1–33 (2006).
18. Pawłowska, K. Katalog zabytków kościanych i rogowych wraz z wynikami analizy archeozoologicznej—stanowisko Giecz [A catalog of bone and antler artifacts with archaeozoological analysis: The Giecz site]. *Museum manuscript of zooarchaeological study* 1–13 (2015).
19. Pawłowska, K. Praktyki depozycyjne w średniowiecznym Gieczu: Perspektywa archeozoologiczna [Depositional practices in medieval Giecz: An zooarchaeological perspective]. *Museum manuscript of zooarchaeological study* (2018).
20. Pawłowska, K. Życie codzienne w zakresie utrzymania i wytwórczości w średniowiecznym Gieczu: Perspektywa archeozoologiczna [Daily life in regards to subsistence and manufacturing in medieval Giecz: A zooarchaeological perspective]. *Museum manuscript of zooarchaeological study* (2022).
21. Pawłowska, K., Miciak, M. & Klimowicz, P. Poverty in medieval Giecz, Poland: A zooarchaeological perspective. *International Council for Archaeozoology, Medieval period Working Group Meeting* (2022).
22. Pawłowska, K., Poradowski, D. & Chrószcz, A. Determining the health condition of Medieval animals using bioarcheological data from Giecz. *International Council for Archaeozoology, Animal Palaeopathology Working Group* (2022).
23. Kubasiewicz, M. Przyczynek do znajomości bydła (Bos Taurus L.) kultury amfor kulistych na ziemiach Polski [A contribution to the knowledge of the cattle (Bos Taurus L.) culture of amphoras in Polish lands]. *Archeol. Polski* **6**, 255–270 (1961).

24. Wiślański, T. Próba wyświetlenia genezy tzw. kultury amfor kulistych. *Archeologia Polski* **8**, 222–245 (1963).
25. Kołodziej, B. Animal burials in the Early Bronze Age in central and eastern Europe. (2010).
26. Szczodrowski, R. Spatial aspects of Globular Amphora Culture funeral rites with animal deposits in Poland. in *The Ritual Killing and Burial of Animals: European Perspectives* (ed. Pluskowski, A.) 51–60 (Oxbow Books, Oxford, England, 2012).
27. Paner, H. The Archaeology of Medieval Gdańsk. in *The Baltic Sea: A Mediterranean of North Europe in the light of Archaeological, Historical and Natural Sciences Research form Ancient to Early Medieval Times.* (ed. Felczak, O.) 101–133 (Scientific Association of Polish Archaeologists Gdańsk Division, Gdańsk, Poland, 2015).
28. Kubasiewicz, M. Szczątki kostne ze stanowiska Gdańsk-Św. Wojciech [Bone remains from the Św. Wojciech site in Gdańsk]. *Prz. Archeol.* **14**, 151–160 (1962).
29. Zalewski, M. Badania nad bezpośrednim zapleczem kopalń krzemieni pasiastych w Krzczonowicach (wyniki prac archeologicznych w rejonie tzw. „Kału Cebuli")[Research on the immediate background of banded flint mines in Krzczonowice (results of archaeological work in the area of the so-called 'kał Cebuli')]. in *Z badań nad wykorzystywaniem krzemienia pasiastego* (ed. Jaskanis, J.) 9–23 (Studia nad gospodarką surowcami krzemiennymi w pradziejach 3, 1996).
30. Jedynak, A. & Kaptur, K. Sprawozdanie z badań osady kultury amfor kulistych na stanowisku 63 w Krzczonowicach, pow. ostrowiecki w roku 2006. *Ostrowieckie zeszyty naukowe* **1**, 19–26 (2008).
31. Jedynak, U., Jedynak, A. & Kaptur, K. Osada ludności kultury amfor kulistych na stanowisku 63 w Krzczonowicach, gm. Ćmielów. Z badań nad zapleczem osadniczym pradziejowych kopalń krzemienia pasiastego [A settlement of the people of the amphorae culture at site 63 in Krzczonowice, Ćmielów municipality. From research on the settlement background of prehistoric banded flint mines]. in *Górnictwo z epoki kamienia: Krzemionki – Polska – Europa. W 90. rocznicę odkrycia kopalni w Krzemionkach* (eds. Piotrowska, D., Piotrowski, W., Kaptur, K. &

Jedynak, A.) 39–58 (Silex et Ferrum, 1, Ostrowiec Świętokrzyski, 2014).

32. Pawłowska, K. Analiza kości zwierzęcych z obiektów kultury łużyckiej w Inowrocławiu (stanowisko 154) [Analysis of animal bones from objects of the Lusatian culture at Inowrocław (site 154)]. in *Inowrocław – st. 154 (AZP 44–40) osada ludności kultury łużyckiej-sprawozdanie z ratowniczych badań archeologicznych w roku 2009 (budowa hali widowiskowo-sportowej przy III LO) [A settlement of the Lusatian culture at Site 154 (AZP 44–40) at Inowrocław: Report on rescue archaeological works carried out in 2009 prior to construction of an entertainment and sports hall at Secondary School No. 3)]* (ed. Woźniak, M.) 1–57 (Archiwum Muzeum im. Jana Kasprowicza, 2011).

33. Pawłowska, K. Depozyty kostne zwierzęce z obiektów okresu przedrzymskiego i rzymskiego w Mosznej Wsi (stanowisko III) [Animal bone deposits from pre-Roman and Roman period features in Moszna Wieś (site III)]. in *Moszna Wieś, gm. Brwinów, woj. mazowieckie, stanowisko III (nr kod. aut. 99), AZP 58–64* 1–11 (IA UW Archives, Warsaw, 2010).

34. Domaradzka, S., Józwiak, B., Machajewski, H. & Waluś, A. Wielokulturowe stanowisko 1 w miejscowości Izdebno Kościelne gmina Grodzisk Mazowiecki: źródła archeologiczne z badań wykopaliskowych na trasie autostrady A2, odcinek mazowiecki (No. 1) [Multicultural site I at Izdebno Kościelne, Grodzisk Mazowiecki]. *Instytut Archeologii Uniwersytetu Warszawskiego* (2016).

35. Pawłowska, K. Osada kultury przeworskiej w Izdebnie Kościelnym. Perspektywa archeozoologiczna w aspekcie rzemieślniczym i depozycyjnym [A settlement of the Przeworsk culture at Izdebno Kościelne: An archaeozoological perspective on craftsmanship and depositional aspects]. in *Wielokulturowe stanowisko I w miejscowości Izdebno Kościelne, gmina Grodzisk Mazowiecki [Multicultural site I at Izdebno Kościelne, Grodzisk Mazowiecki]* (eds. Domaradzka, S., Józwiak, B., Machajewski, H. & Waluś, A.) vol. 1 393–417 (VIA Archaeologica Masoviensis, Światowit Suplement Series M, 2016).

36. Hansen, S. *et al.* Pietrele am 'Lacul Gorgana': Bericht über die Ausgrabungen in der

neolithischen und kupferzeitlichen Siedlung und die geomorphologischen Untersuchungen in den Sommern 2012–2016. *Eurasia antiqua: Zeitschrift für Archäologie Eurasiens* 1–116 (2014).

37. Hansen, S. Arbeitsteilung, soziale Ungleichheit und Surplus in der Kupferzeit an der Unteren Donau 4600–4300 v. Chr. in *Überschuss ohne Staat – Politische Formen in der Vorgeschichte. Surplus without the State – Political Forms in Prehistory: 10. Mitteldeutscher Archäologentag vom 19. bis 21. Oktober 2017 in Halle (Saale)* (eds. Meller, H., Gronenborn, D. & Risch, R.) 221–245 (Tagungen des Landesmuseums für Vorgeschichte Halle Bd. 18, Halle (Saale), 2018).
38. Ritchie, K. Copper and water: aquatic resourcesin the Chalcolithic of south-eastern Europe. in *Interaktion ohne Grenzen. Beispiele archäologischer Forschungen am Beginn des 21. Jahrhunderts* (eds. Eriksen, B. V., Abegg-Wigg, A., Bleile, R. & Ickerodt, U.) vol. 1 71–77 (Stiftung Schleswig-Holsteinische Landesmuseen, Schleswig, 2017).
39. Benecke, N. *et al.* Pietrele in the Lower Danube region: integrating archaeological, faunal and environmental investigations. *Doc. Praehist.* **40**, 175–193 (2013).
40. Benecke, N. Archäozoologische Untersuchungen. in *Bericht über die Ausgrabungen in der kupferzeitlichen Tellsiedlung Ma˘gura Gorga- na bei Pietrele in Muntenien/Rumänien im Jahre 2002* (eds. Hansen, S. et al.) vol. 10 1–53 (Eurasia antiqua, 2004).
41. Benecke, N. Archäozoologische Untersuchungen. in *Pietrele – Eine kupferzeitliche Siedlung an der Unteren Donau. Bericht über die Ausgrabung im Sommer 2005* (eds. Hansen, S. et al.) vol. 12 54–57 (Eurasia Antiqua, 2006).
42. Korenevskij, S. N., Belinskij, A. B. & E., K. A. A. Bol'šoj Ipatovskij kurgan na Stavropol'e kak arheologičeskij istočnik po èpohe bronzovogo veka na stepnoj granice Vostočnoj Evropy i Kavkaza. (2007).
43. Wang, C.-C. *et al.* Ancient human genome-wide data from a 3000-year interval in the Caucasus corresponds with eco-geographic regions. *Nat. Commun.* **10**, 590 (2019).
44. Key, F. M. *et al.* Emergence of human-adapted Salmonella enterica is linked to the Neolithization process. *Nature ecology & evolution* **4**, 324–333 (2020).

45. Siliézar, A. *et al.* Analysen stabiler Isotope zur Ernährungsrekonstruktion von bronzezeitlichen Individuen aus Kurgan 2, Ipatovo, Nordkaukasus, Russland. *Bulletin der Schweizerischen Gesellschaft für Anthropologie* **24**, 3–17 (2018).

46. Scott, A. *et al.* Emergence and intensification of dairying in the Caucasus and Eurasian steppes. *Nat. Ecol. Evol.* **6**, 813–822 (2022).

47. Ghalichi, A. *et al.* The rise and transformation of Bronze Age pastoralists in the Caucasus. *Nature* **635**, 917–925 (2024).

48. Reinhold, S. *et al.* Contextualising innovation. Cattle owners and wagon drivers in the North Caucasus and beyond. in *Appropriating Innovations: Entangled Knowledge in Eurasia, 5000-1500 BCE* (eds. Stockhammer, P. W. & Maran, J.) 78–97 (Oxbow Books, Oxford, England, 2017).

49. Kantorovich, A. R., Maslov, V. E. & Petrenko, V. G. Pogrebeniya maykopskoy kultury kurgana No. 1 mogilnika Marinskaya 5. *Materialy po izucheniyu istoriko-kulturnogo naslediya Severnogo Kavkaza* **10**, 71–108 (2013).

50. Reinhold, S. & Belinskiy, A. B. »Neue« Orte für eine »neue« Kultur: ein nicht-koloniales Interaktionszenarium im Hochgebirge des Kaukasus am Beginn der Spätbronzezeit. in *Kontaktmodi. Ergebnisse der gemeinsamen Treffen der Arbeitsgruppen 'Mobilität und Migration' und 'Zonen der Interaktion'* (eds. Marzoli, D., Reinhold, S., Schlotzhauer, U., Vogt, B. & Schnorbusch, H.) 115–138 (Harrassowitz Verlag, 2020).

51. Reinhold, S. *et al.* At the onset of settled pastoralism – Implications of archaeozoological and isotope analyses from Bronze age sites in the North Caucasus. *Quat. Int.* **700-701**, 50–67 (2024).

52. Berdiev, O. Mondzhukly-depe—mnogosloynoe poselenie neolita i rannego eneolita v yuzhnom Turkmenistane [Monjukli Depe—a multi-layered Neolithic and Early Aeneolithic settlement in southern Turkmenistan]. *Karakumskie Drevnosti* **4**, 11–34 (1972).

53. Bernbeck, R. & Pollock, S. Scalar differences: temporal rhythms and spatial patterns at Monjukli Depe, southern Turkmenistan. *Antiquity* **90**, 64–80 (2016).

54. *Looking Closely: Excavations at Monjukli Depe, Turkmenistan, 2010 - 2014*. (Sidestone Press, Leiden, Netherlands, 2019).

55. Benecke, N. Archaeozoological Investigations. in *Excavations at Monjukli Depe, Meana-Čaača Region, Turkmenistan, 2010* (eds. Pollock, S. et al.) vol. 43 169–237 (Archäologische Mitteilungen aus Iran und Turan, 2011).

56. Benecke, N. The Fauna of Monjukli Depe – Environmental implications. in *Archaeological work at Monjukli Depe: a regional perspective* (eds. Pollock, S. et al.) vol. 47 1–47 (Archäologische Mitteilungen aus Iran und Turan, 2018).

57. Eger, J. Remains of the Feast Days? A Comparative Study of Faunal Remains from Aeneolithic Monjukli Depe. in *Looking closely: Excavations at monjukli Depe, Turkmenistan, 2010 - 2014* (eds. Pollock, S., Bernbeck, R. & Öğüt, B.) (Sidestone Press, Leiden, Netherlands, 2019).

58. Eger, J. *Mensch-Tier-Verhaltnisse in Monjukli Depe: Eine Analyse des sozialen Zusammenlebens in einer neolithisch-aneolithischen Siedlung in Turkmenistan*. (Sidestone Press, Leiden, Netherlands, 2022).

59. Eger, J., Knipper, C. & Benecke, N. Stable isotope evidence for animal husbandry practices at prehistoric monjukli Depe, southern Turkmenistan. in *Archaeozoology of Southwest Asia and Adjacent Areas XIII* (ed. Daujat, J., Hadjikoumis, A., Berthon, R., Chahoud, J., Kassianidou, V., Vigne, J.-D.) 41–60 (Lockwood Press, Atlanta, GA, 2021). doi:10.5913/aswaxiii.0130103.

60. Kaniuth, K. The Late Bronze Age settlement of Tilla Bulak (Uzbekistan): A summary of four years' work. in *South Asian Art and Archaeology 2012: Man and Environment in Prehistoric and Protohistoric South Asia: New Perspectives* (eds. Lefèvre, V., Didier, A. & Mutin, B.) 117–128 (Brepols Turnhout, Belgium, 2016).

61. Kaniuth, K. Life in the countryside: The rural archaeology of the Sapalli culture. in *The World of the Oxus Civilization* (eds. Lyonnet, B. & Dubova, N.) 457–486 (Taylor & Francis, London, England, 2021).

62. Kaniuth, K. Tilla Bulak 2009 – Vorbericht zur dritten Kampagne. *Archäologische Mitteilungen aus*

Iran und Turan **42**, 129–163 (2010).
