# Supplementary material for: Probing the zooarchaeological record across time and space for ancient pathogen DNA
Source: Nat Commun. 2026 Apr 30;17:3469. doi: 10.1038/s41467-026-71543-4 (PMC13133277; doi:10.1038/s41467-026-71543-4)
Supplement: Supplementary file 3 — Description of Additional Supplementary Files [file 41467_2026_71543_MOESM3_ESM.pdf]

Description of Additional Supplementary Files

File Name: Supplementary Data 1
Description: Archaeological sites included in this study. Age is BCE unless stated otherwise.

File Name: Supplementary Data 2
Description: Archaeological samples included in the study. Taxonomic classification is resolved based on the morphological assignment and, when DNA preservation was sufficient, on the recovered host DNA. Samples labelled with ≥0.1% blue & ≥1% green background. Samples with insufficient host DNA recovery (<0.1%) marked in grey. DS: Dual strand. SS: Single strand.

File Name: Supplementary Data 3
Description: List of taxa included in the pathogen screening.

File Name: Supplementary Data 4
Description: Samples used for phylogenetic reconstructions of E. rhusiopathiae and S. lutetiensis. Accessions gives the raw sequencing reads, or the GenBank/RefSeq accession for the assemblies of samples used when no raw sequencing reads were available. When GenBank/Refseq accessions given (GCA/GCF), simulated sequencing reads were generated using wgsim and remapped to the reference genome. QC pass into tree designates if a sample failed to pass basic QC (minimum coverage on unfiltered variant sites >4x) for modern samples. Samples which failed QC were not utilized for identifying phylogenetically informative positions and were not included in the phylogenetic reconstruction. For ancient samples, projections were carried out if the average coverage on unfiltered variant sites was >0.05x. Included for SNP identification column indicates whether the sample passed QC for being considered for identification of phylogenetic informative sites.

File Name: Supplementary Data 5
Description: Mapping statistics and variant coverage statistics for ancient samples passing QC to be projected or called (ldo050) in phylogenetic trees. Phylogenetic trees were produced by a SNP table using RAxML-ng across sites which were variable among modern representative genomes and called across all ingroup modern samples. For E. rhusiopathiae, the ancient sample ldo050 was also allowed to identify variant sites. The total number of variant sites used for phylogenetic reconstruciton of E. rhusiopathiae was 11,970 sites. The total number of variant sites used for phylogenetic reconstruction of S. lutetiensis was 92,526 sites. All ancient samples had 3 terminal bases masked following mapping prior to variant calling, to reduce the impacts of aDNA damage in phylogenetic reconstruction. Reported values for reads_mapped, genome_coverage, genome_percent_breadth and frequency_damage_5_prime_terminal_base are calculated on deduplicated, unfiltered bam files post mapping, prior to any quality filtering or terminal base masking.
